# Quantitative proteomics in A30P*A53T α-synuclein transgenic mice reveals upregulation of Sel1l

**DOI:** 10.1371/journal.pone.0182092

**Published:** 2017-08-03

**Authors:** Jianguo Yan, Pei Zhang, Fengjuan Jiao, Qingzhi Wang, Feng He, Qian Zhang, Zheng Zhang, Zexi Lv, Xiang Peng, Hongwei Cai, Bo Tian

**Affiliations:** 1 Department of Neurobiology, Tongji Medical School, Huazhong University of Science and Technology, Wuhan, Hubei Province, P. R. China; 2 Key Laboratory of Neurological Diseases, Ministry of Education, Wuhan, Hubei Province, P. R. China; 3 Institute for Brain Research, Collaborative Innovation Center for Brain Science, Huazhong University of Science and Technology, Wuhan, Hubei Province, P. R. China; Florey Institute of Neuroscience and Mental Health, The University of Melbourne, AUSTRALIA

## Abstract

α-Synuclein is an abundantly expressed neuronal protein that is at the center of focus in understanding a group of neurodegenerative disorders called synucleinopathies, which are characterized by the intracellular presence of aggregated α-synuclein. However, the mechanism of α-synuclein biology in synucleinopathies pathogenesis is not fully understood. In this study, mice overexpressing human A30P*A53T α-synuclein were evaluated by a motor behavior test and count of TH-positive neurons, and then two-dimensional liquid chromatography-tandem mass spectrometry coupled with tandem mass tags (TMTs) labeling was employed to quantitatively identify the differentially expressed proteins of substantia nigra pars compacta (SNpc) tissue samples that were obtained from the α-synuclein transgenic mice and wild type controls. The number of SNpc dopaminergic neurons and the motor behavior were unchanged in A30P*A53T transgenic mice at the age of 6 months. Of the 4,715 proteins identified by proteomic techniques, 271 were differentially expressed, including 249 upregulated and 22 downregulated proteins. These alterations were primarily associated with mitochondrial dysfunction, oxidative stress, ubiquitin-proteasome system impairment, and endoplasmic reticulum (ER) stress. Some obviously changed proteins, which were validated by western blotting and immunofluorescence staining, including Sel1l and Sdhc, may be involved in the α-synuclein pathologies of synucleinopathies. A biological pathway analysis of common related proteins showed that the proteins were linked to a total of 31 KEGG pathways. Our findings suggest that these identified proteins may serve as novel therapeutic targets for synucleinopathies.

## Introduction

In the past two decades, α-synuclein has been the center of focus in understanding the etiology of a group of overlapping neurodegenerative disorders called synucleinopathies, which include Parkinson’s disease (PD), Parkinson’s disease dementia (PDD), dementia with Lewy bodies (DLB), multiple system atrophy (MSA) and a number of less well-characterized neuroaxonal dystrophies[1–3]. The universal feature of α-synucleinopathies is the presence of proteinaceous intracellular bodies containing aggregates of α-synuclein[1–3]. However, the mechanisms that underlie the aberrant functions of α-synuclein and how these impact on disease pathogenesis remain poorly understood. The numerous murine transgenic lines overexpressing human WT, A53T, or A30P mutant α-synuclein develop synucleinopathy, neurodegeneration, loss of striatal dopamine, and locomotor dysfunction, which also result in mitochondrial dysfunction, oxidative stress, and activation of cell death pathways[4–6]. Therefore, our understanding of the importance of α-synuclein biology in synucleinopathies pathogenesis has grown considerably. The overexpression of human A30P*A53T α-synuclein in mice is used in PD research[7–9]. The number of SNpc dopaminergic neurons and levels of dopamine were unchanged in A30P*A53T transgenic mice up to 9 months old[7], but significantly decreased levels of dopamine and motor impairment were recognized at 16 months old[8]. Therefore, the A30P*A53T α-synuclein transgenic mouse model is a useful model for analyzing the pathological cascade from aggregated α-synuclein to motor disturbance.

Proteomics is a powerful methodology to investigate how protein expression is affected in the pathogenesis of a disease process, providing a complement to the information obtained by functional genomics. Such unbiased approaches permit the identification of novel protein changes and are used to study various illnesses[10, 11]. Quantitative proteomics can be performed using three methods: label-free quantification, metabolic labeling with stable isotope labeling by amino acids in cell culture, and stable-isotope labeling using chemical reagents covalently attached in vitro such as dimethyl-labeling, tandem mass tags (TMTs), and isobaric tags for relative and absolute quantification (iTRAQ)[10, 11]. Notably, the use of isobaric tag-based TMTs and iTRAQ produces high-quality data with high sensitivity, excellent signal-to-noise ratios, and a broad dynamic range. Owing to their many advantages, TMTs and iTRAQ have gained popularity as essential tools for quantitative proteomics[10, 11].

To gain insight into the mechanism of α-synuclein pathologies, we used TMTs to generate comparative protein profiles of SNpc samples obtained from A30P*A53T α-synuclein transgenic mice and controls at the age of 6 months. We compared the SNpc tissue levels of candidate proteins to evaluate their ability to discriminate between α-synuclein transgenic and control mice. Our findings indicate that proteomics is a useful method to investigate the crucial pathogenesis of α-synucleinopathies.

## Materials and methods

### Animals

To establish a transgenic PD mouse model, C57BL/6J-Tg (Th-SNCA*A30P*A53T) 39Eric/J transgenic mice (Stock number: 008239) were purchased from the Jackson Laboratory. To maintain the α-synuclein-A30P*A53T transgenic (TG) mice in our laboratory, established transgenic mice were mated with wild-type C57BL/6J background (Beijing Vital River Laboratory Animal Technology Co., Ltd, Beijing, China). The offspring were genotyped by tail-tip DNA PCR according to the genotyping protocols database of the Jackson Laboratory website. Theα-synuclein-A30P*A53T transgenic mice were genotyped with 4 primers: Transgene -F, 5'-CAG GTA CCG ACA GTT GTG GTG TAA AGG AAT-3', Transgene-R, 5'-GAT AGC TAT AAG GCT TCA GGT TCG TAG TCT-3', and Internal Positive Control-F, 5'-CAA ATG TTG CTT GTC TGG TG-3', Internal Positive Control-R, 5'-GTC AGT CGA GTG CAC AGT TT-3' (Transgene = 469bp, Wild type = no bands, Internal Positive Control = 200bp). The cycling conditions were as follows: 97°C for 3min, (97°C for 30 s, 65°C for 30s and 72°C for 30 s) ×35, followed by 2 min at 72°C. The transgenic mice and their wild-type littermates were investigated at the age of 6 months with a motor behavior test, TH-positive neurons count and proteomic analyses. Animals were housed under standard housing conditions with a 12h light/12h dark cycle and unlimited access to water and chow. The protocol was approved by the Animal Care and Use Committee of Huazhong University of Science and Technology.

### Rotarod test

The rotarod test was performed to assess motor coordination by placing mice on a rotating rod that runs at an accelerating speed from 3 to 30 rpm over a 5-min period. If a mouse falls onto an underlying platform, then the detector automatically stops and records the fall down latency. Prior to the first test, the mice were trained with three trials, with the rod rotating at a constant speed of 3 rpm for approximately one minute.

### Grip strength test

The forelimb grip strength test was used to evaluate the muscle strength or neuromuscular activity in mice. The mice were held up to grip the pull bar on the grip wire with only their front paws and were steadily pulled back until they could not hold on any longer. This ability was measured 10 times per mouse, and the maximal force (in grams) was recorded.

### Open field test

The exploration and reactivity of mice to a novel space was assessed in a Plexiglas cage (45cm × 45cm). Mice were placed individually into the center of a brightly lit open field. Their movements were tracked for the ensuing 20 min. All spontaneous locomotion of the mice was measured by an activity monitor connected to a computer.

### Immunofluorescence staining

After being anesthetized, the brains were removed from the skull, and fixed in 4% paraformaldehyde overnight, and then stored at 4°C in 30% sucrose solution until they sank. The brains were freeze sectioned using a sliding microtome (Leica, Germany) into 30-μm coronal sections. For immunofluorescence staining, the slices were permeabilized in 0.3% triton for 10 min, and then blocked with 10% serum in PBS for 1 h and incubated with a primary antibody (TH, Santa Cruz, sc-374047, 1:200; Sel1l, Sangon Biotech, D161115, 1:200; Sdhc, Proteintech Group, 14575-1-AP, 1:100) overnight at 4°C. The next day, the secondary antibodies (Dylight 594-Conjugated AffiniPure Goat Anti-Mouse, Jackson ImmunoResearch, 1:400; Dylight 488-Conjugated AffiniPure Goat Anti-Rabbit IgG, Jackson ImmunoResearch, 1:400) were added to the sections. Nuclei were stained by DAPI. The slices were imaged using microscopy (Leica DFC320, Germany). The number of TH-immunoreactive positive neurons in the SNpc was counted using the previously described counting criteria[12].

### Protein extraction and trypsin digestion

The SNpc tissues of the transgenic mice and their wild-type littermates were removed at the age of 6 months, and the samples were frozen immediately in liquid nitrogen. The samples were transferred to 5-mL centrifuge tubes and lysed in buffer (8 M urea, 2 mM EDTA and 1% Protease Inhibitor Cocktail) and then sonicated three times on ice, and centrifuged at 20,000g at 4°C for 10 min to remove cellular debris. Finally, the protein was precipitated with cold 15% TCA (trichloroacetic acid, Sigma) for 2 h at -20°C. After centrifugation at 4°C for 10 min, the remaining precipitate was washed with cold acetone three times. Proteins were resuspended in buffer (8 M urea, 100 mM TEAB, pH 8.0), and the protein concentrations were determined using a 2-D Quant kit according to the manufacturer's instructions. For trypsin digestion, the protein solution was reduced in 10 mM DTT (dithiothreitol, Sigma) for 60 min at 37°C, alkylated in 20 mM IAA (iodoacetamide, Sigma) for 45 min at 37°C in the dark and digested overnight by trypsin (trypsin/protein mass ratio 1:50). Approximately 100 μg of protein for each sample was digested with trypsin for the following experiments.

### TMT labeling

After trypsin digestion, peptide was desalted by a Strata X C18 SPE column (Phenomenex) and vacuum-dried. Peptide was reconstituted in 0.5 M TEAB and processed according to the manufacturer's protocol for the 6-plex TMT kit (Thermo Fisher Scientific). Briefly, one unit of TMT reagent (defined as the amount of reagent required to label 100 μg of protein) was thawed and reconstituted in 24 μl CAN (acetonitrile, Fisher Chemical). The peptide mixtures were then incubated for 2 h at 25°C and pooled, desalted using C18 reversed-phase spin columns according to the manufacturer’s instructions and dried by vacuum centrifugation.

### Quantitative proteomic analysis by LC-MS/MS

The sample was then fractionated into fractions by high pH reverse-phase HPLC using Agilent 300Extend C18 column. Briefly, peptides were first separated with a gradient of 2% to 60% acetonitrile in 10 mM ammonium bicarbonate pH 10 over 80 min into 80 fractions. Then, the peptides were combined into 18 fractions and dried under vacuum. Peptides were dissolved in 0.1% FA (Formic acid, Fluka) and directly loaded onto a reversed-phase pre-column (Acclaim PepMap 100, Thermo Scientific). Peptide separation was performed using a reversed-phase analytical column (Acclaim PepMap RSLC, Thermo Scientific). The gradient was composed of an increase from 6% to 85% solvent B (0.1% FA in 98% ACN), all at a constant flow rate of 300 nl/min on an EASY-nLC 1000 UPLC system. The resulting peptides were analyzed by a Q Exactive^™^ Plus hybrid quadrupole-Orbitrap mass spectrometer (ThermoFisher Scientific).The peptides were subjected to NSI source followed by tandem mass spectrometry (MS/MS) in Q Exactive^™^ Plus (Thermo) coupled online to the UPLC. Intact peptides were detected in the Orbitrap at a resolution of 70,000. Peptides were selected for MS/MS using the NCE setting at 27, 30, 33; ion fragments were detected in the Orbitrap at a resolution of 17,500. A data-dependent procedure that alternated between one MS scan followed by 20 MS/MS scans was applied for the top 20 precursor ions above a threshold ion count of 2.0E4 in the MS survey scan with a 30.0-s dynamic exclusion.

The resulting MS/MS data were processed using Mascot search engine (v.2.3.0). Tandem mass spectra were searched against the SwissProt_Mouse database. Trypsin/P was specified as cleavage enzyme allowing up to 2 missing cleavages. The mass error was set to 10 ppm for precursor ions and 0.02 Da for fragment ions. Carbamidomethyl on Cys was specified as a fixed modification and oxidation on Met was specified as a variable modification. For the protein quantification method, TMT-6plex was selected in Mascot. FDR was adjusted to < 1%, and the peptide ion score was set > 20.

### Bioinformatics analysis

Some identified proteins that appear in some samples but not in others were removed. Gene Ontology (GO) annotation of the proteome was performed using the UniProt-GOA database (http://www.ebi.ac.uk/GOA/). Cluster membership was visualized by a heat map using the "heatmap.2" function from the "gplots" R-package. There, we used wolfpsort a subcellular localization prediction software, to predict subcellular localization. If some identified proteins were not annotated by the UniProt-GOA database, then the InterProScan software was used to annotate the protein's GO function based on the protein sequence alignment method. Proteins were classified by Gene Ontology annotation based on three categories: biological process, cellular component and molecular function. The Kyoto Encyclopedia of Genes and Genomes (KEGG) database was used to annotate the protein KEGG pathway by a two-tailed Fisher's exact test.

### Western blot

The substantia nigra tissues of mice were homogenized in ice-cold RIPA buffer with protease inhibitors. The lysates were centrifuged at 12000 g for 15 min at 4°C, and the supernatant fractions were collected. The supernatant fractions were re-suspended in 1 × SDS sample buffer and boiled at 98°C for 10 min. Samples were separated on 12% SDS-PAGE gels and transferred to a PVDF membrane and then sequentially incubated with the primary (β-actin, Cell Signaling Technology, 8457, 1:5000; Sel1l, 1:2000; Sdhc, 1:1000) and secondary (Peroxidase AffiniPure Goat Anti-Mouse IgG, Jackson ImmunoResearch, WB-1:20000; Peroxidase AffiniPure Goat Anti-Rabbit IgG, Jackson ImmunoResearch, WB-1:20000) antibodies. The detection was performed using the ECL western blotting detection system.

### ELISA

Plasma samples were collected using heparin as an anticoagulant. Centrifugation was for 15 minutes at 1000 g at 4°C. ELISA kits (Sel1l, Cusabio, CSB-EL020973; Sdhc, Cloud-clone Corp, SEK213) were utilized to quantify Sel1l and Sdhc in plasma. Samples and standards were prepared according to the instructions from the manufacturer.

### Statistical analysis

All data were analyzed by two-tailed unpaired t-tests. All data are presented as the mean ± SD. All results are representative of at least three independent experiments. P values less than 0.05 were considered to be statistically significant.

## Results

### Motor behavior and dopaminergic neurons were detected in the transgenic mice and their wild-type littermates

**Fig 1:**
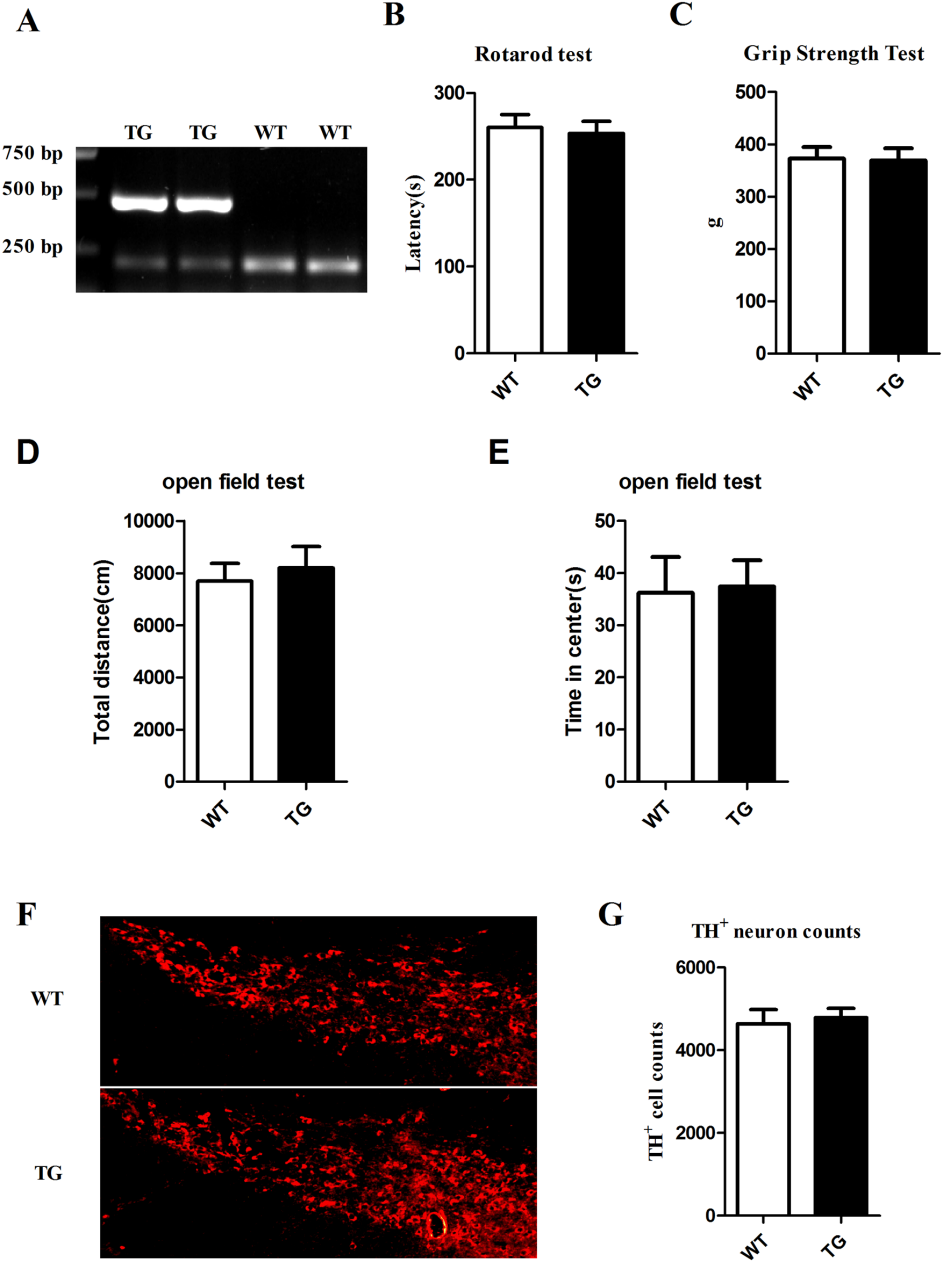
Behavioral and immunohistochemical analysis of transgenic mice and their wild-type littermates. (A) RT-PCR was used to verify the mRNA expression of α-synuclein-A30P*A53T transgenic mice (TG) and wild-type littermates (WT). (B) The rotarod test was used to measure TG mice and WT controls at the age of 6 months (n = 15). (C) Grip strength tests were performed in TG mice and WT controls (n = 15). (D and E) Open field tests were performed in TG mice and WT controls (n = 15). (F) Immunostaining of tyrosine hydroxylase (TH)-positive neurons of SNpc in TG mice and WT controls. (G) The number of TH-immunoreactive positive neurons in the SNpc was counted stereologically (n = 3).

A30P*A53T transgenic mice from Jackson Laboratory were bred, and the offspring were genotyped by tail-tip DNA PCR (Fig 1A). The motor behavior test and counting of TH-positive neurons within SNpc were performed in the A30P*A53T transgenic mice and their wild-type littermates at the age of 6 months. Compared to the WT mice, the A30P*A53T transgenic mice displayed no distinction in PD-related behavior, such as in the rotarod test (Fig 1B), grip strength test (Fig 1C), open field test (Fig 1D and 1E), and TH positive neurons numbers within SNpc (Fig 1F and 1G). These results indicate that A30P*A53T transgenic mice at the age of 6 months show no obvious symptoms of PD in behavior phenotype or DA neuron loss.

### TMT proteomics profiling of the SNpc of α-synuclein A30P*A53T transgenic and WT mice

**Fig 2:**
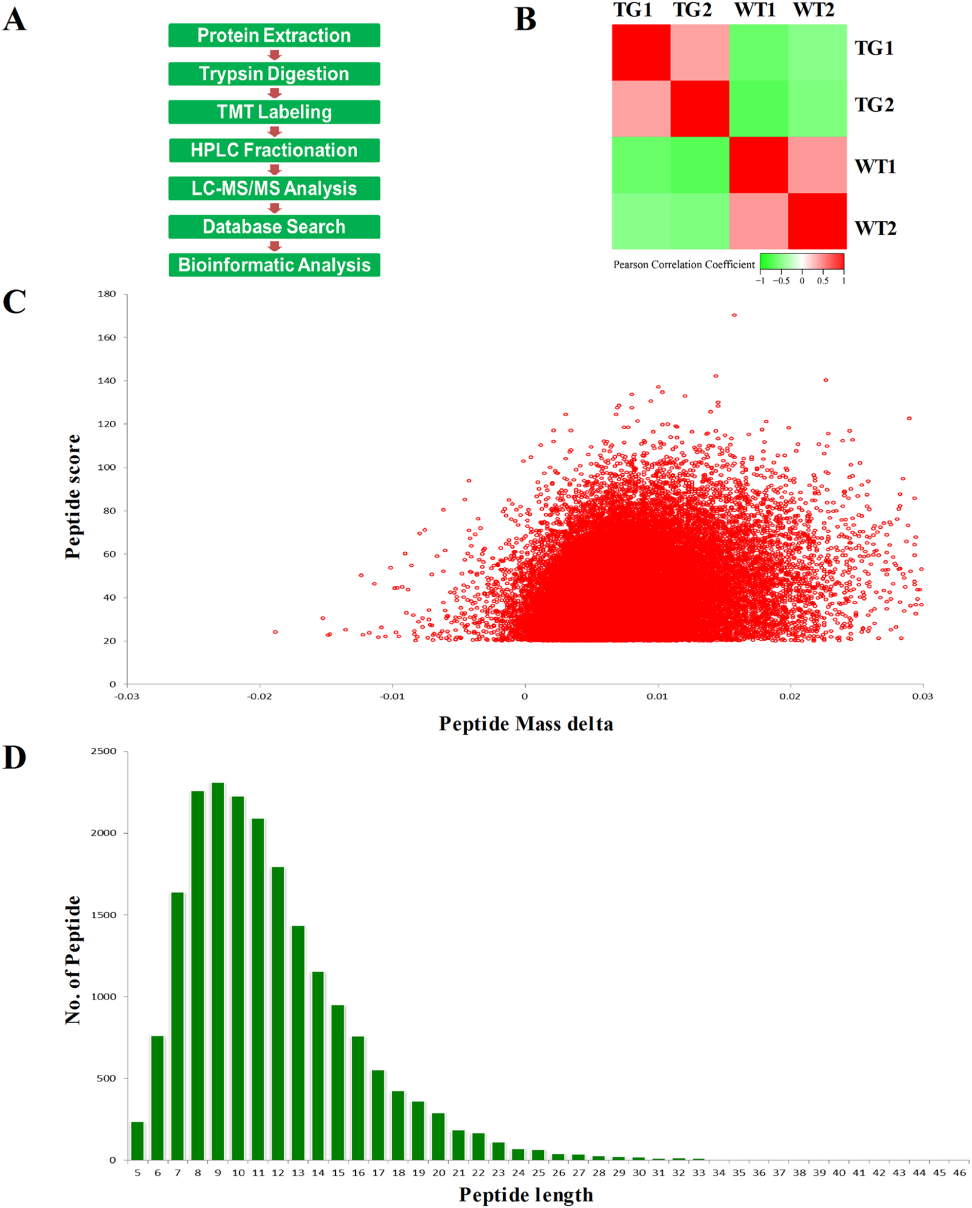
Proteomic process flow chart and standardized sample evaluation. (A) Depiction of the experimental design workflow. (B) Data reproducibility reflected by Pearson correlation coefficients. (C) Mass error distribution of all identified peptides. (D) Peptide length distribution.

**Fig 3:**
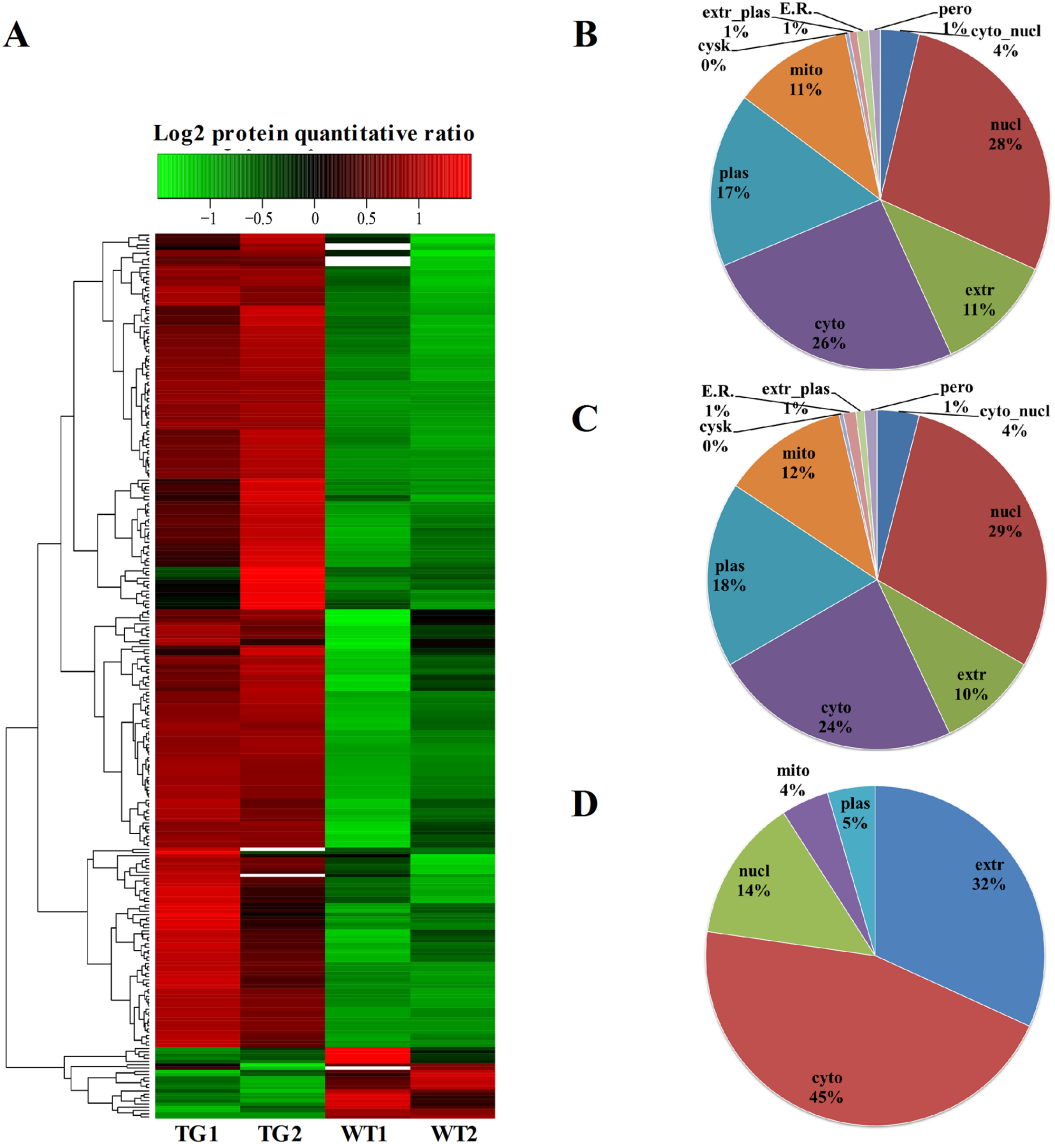
Heatmap and the subcellular locations of proteins. (A) Heatmap of proteins identified as significantly differentially regulated (*p* < 0.05). (B) Subcellular locations of all identified proteins. (C) Subcellular locations of upregulated proteins. (D) Subcellular locations of downregulated proteins.

The SNpc of 6-month-old A30P*A53T transgenic mice (n = 40) and WT littermates (n = 40) was extracted and randomly divided into two samples: TG1 and TG2; WT1 and WT2. Samples were then subjected to a high-throughput quantitative proteome analysis using TMTs (Fig 2A). Pearson correlation coefficients of all samples were used to evaluate the repeatability of the relative protein quantitation (Fig 2B). MS data validation is shown in Fi 2C and 2D. First, we assessed the mass error of all identified peptides (Fig 2C). The distribution of mass error was near zero, and many were <0.02 Da; thus, the mass accuracy of the MS data was acceptable. Second, the length of most peptides was distributed between 8 and 16, similar to tryptic peptides (Fig 2D). Thus, the sample preparation was markedly better than that of the standards. A total of 4,715 protein groups were identified, and 3,450 of these were quantified in the SNpc of A30P*A53T transgenic mice and WT littermates (S1 Table). The quantified proteins were divided into two categories. A ratio >1.2 was considered upregulation, and <0.83 was considered downregulation. Among the quantified proteins, 249 were upregulated, and 22 were downregulated (S1 Table). Fig 3A shows a heatmap of the abundance patterns for proteins with significant changes in the SNpc of A30P*A53T mice and WT littermates. Based on the subcellular location annotation information of identified proteins, we counted the amount and percentage of differentially expressed proteins. Among these, some were found in one or more locations within the cell: 26% were in the cytoplasm, 28% in the nucleus, 17% in the plasma membrane, 11% in mitochondria, and 11% in the extracellular matrix (ECM) (Fig 3B). Among the 249 upregulated proteins, 24% were in the cytoplasm, 29% in the nucleus, 18% in the plasma membrane, 12% in mitochondria, and 10% in the ECM (Fig 3C). Among the 22 downregulated proteins, 45% were in the cytoplasm, 14% in the nucleus, 5% in the plasma membrane, 4% in the mitochondria, and 32% in the ECM (Fig 3D).

### GO term classification and GO functional enrichment of differentially quantified proteins

To assess the nigral proteome composition between A30P*A53T transgenic mice and WT controls, differentially quantified proteins were parsed into GO term classifications and GO functional enrichment. GO annotation covers three domains: biological process, cellular components, and molecular function. Per the biological process classification, most of these 249 upregulated proteins were involved in various cellular processes, such as biological regulation, response to stimulus, and metabolic process (S2 Table); most of these 22 down-regulated proteins were involved in various cellular processes, such as biological regulation, response to stimulus, and metabolic process (S3 Table). Per the cellular component classification, most of the 249 upregulated proteins were found to be localized virtually everywhere in the cells and even in the ECM, such as the organelle, membrane, cell junction, or extracellular region (S2 Table), and most of the 22 downregulated proteins were found to be localized virtually everywhere in the cells and even in the ECM, such as the organelle, membrane or extracellular region (S3 Table). Based on the molecular function classification, most of the 249 upregulated protein functions were binding, catalytic activity, transporter activity, and enzyme regulator activity (S2 Table), and most of the 22 downregulated protein functions were binding, catalytic activity, enzyme regulator activity, transporter activity, and antioxidant activity (S3 Table).

**Fig 4:**
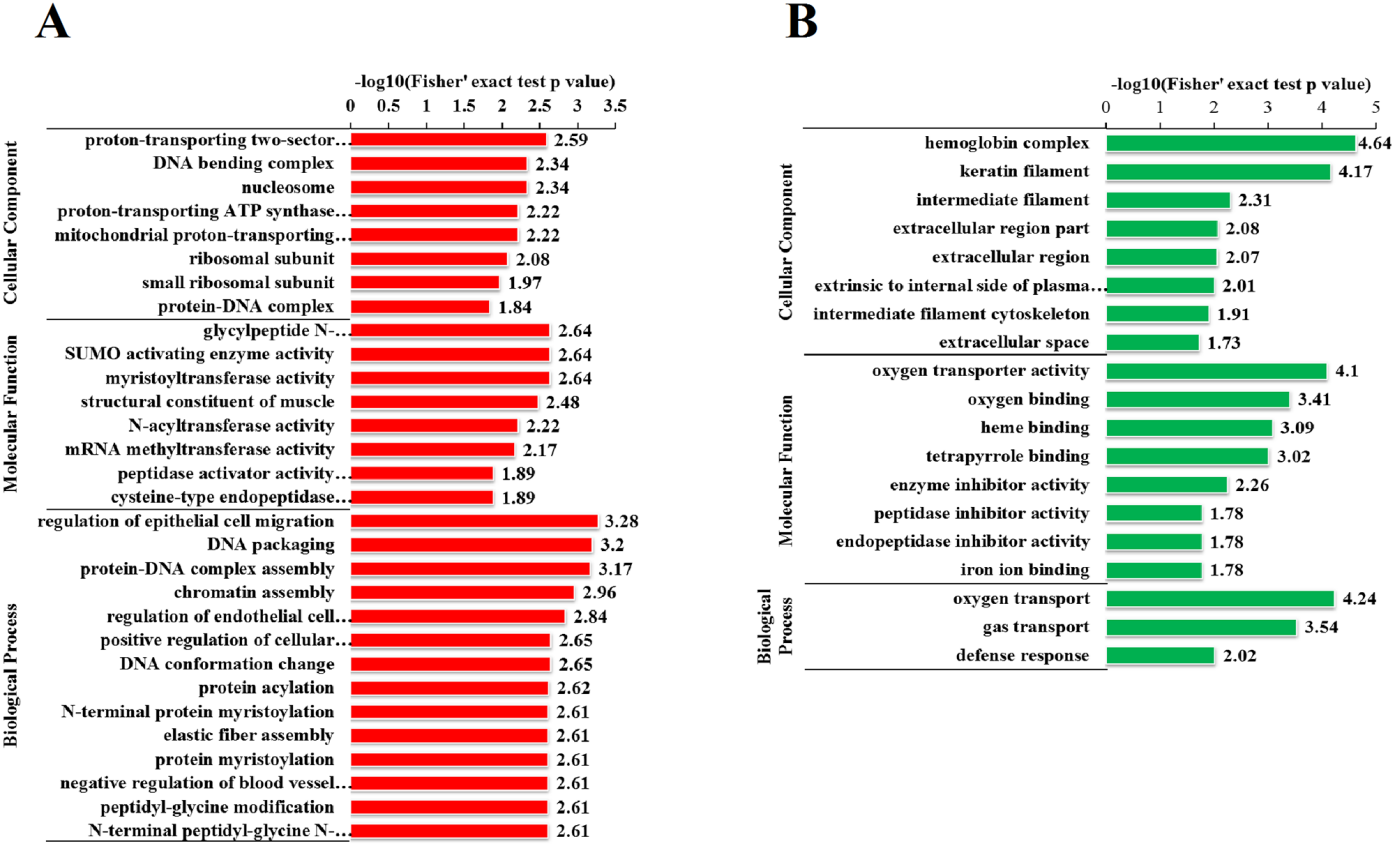
GO-based enrichment analysis. (A) GO-based enrichment analysis of upregulated proteins. (B) GO-based enrichment analysis of down-regulated proteins.

Per the biological process of GO functional enrichment, most of the 249 upregulated proteins were involved in the regulation of epithelial cell migration, DNA packaging, protein-DNA complex assembly, and chromatin assembly (Fig 4A), and most of the 22 downregulated proteins were involved in oxygen transport, gas transport, and defense response (Fig 4B). Per the cellular component of GO functional enrichment, most of the 249 upregulated proteins were proton-transporting two-sector ATPase complex, DNA-binding complex, or mitochondrial proton-transporting ATP synthase (Fig 4A), and most of the 22 downregulated proteins were a part of the hemoglobin complex, keratin filament, intermediate filament, or extracellular region (Fig 4B). Per the molecular function of GO functional enrichment, most of the 249 upregulated proteins showed glycylpeptide N-tetradecanoyltransferase activity, SUMO-activating enzyme activity, N-acyltransferase activity, or peptidase activator activity involved in apoptosis (Fig 4A), and most of the 22 downregulated proteins showed oxygen transporter activity, oxygen binding, heme binding, or tetrapyrrole binding (Fig 4B).

### KEGG pathway enrichment analysis of differentially expressed proteins

**Fig 5:**
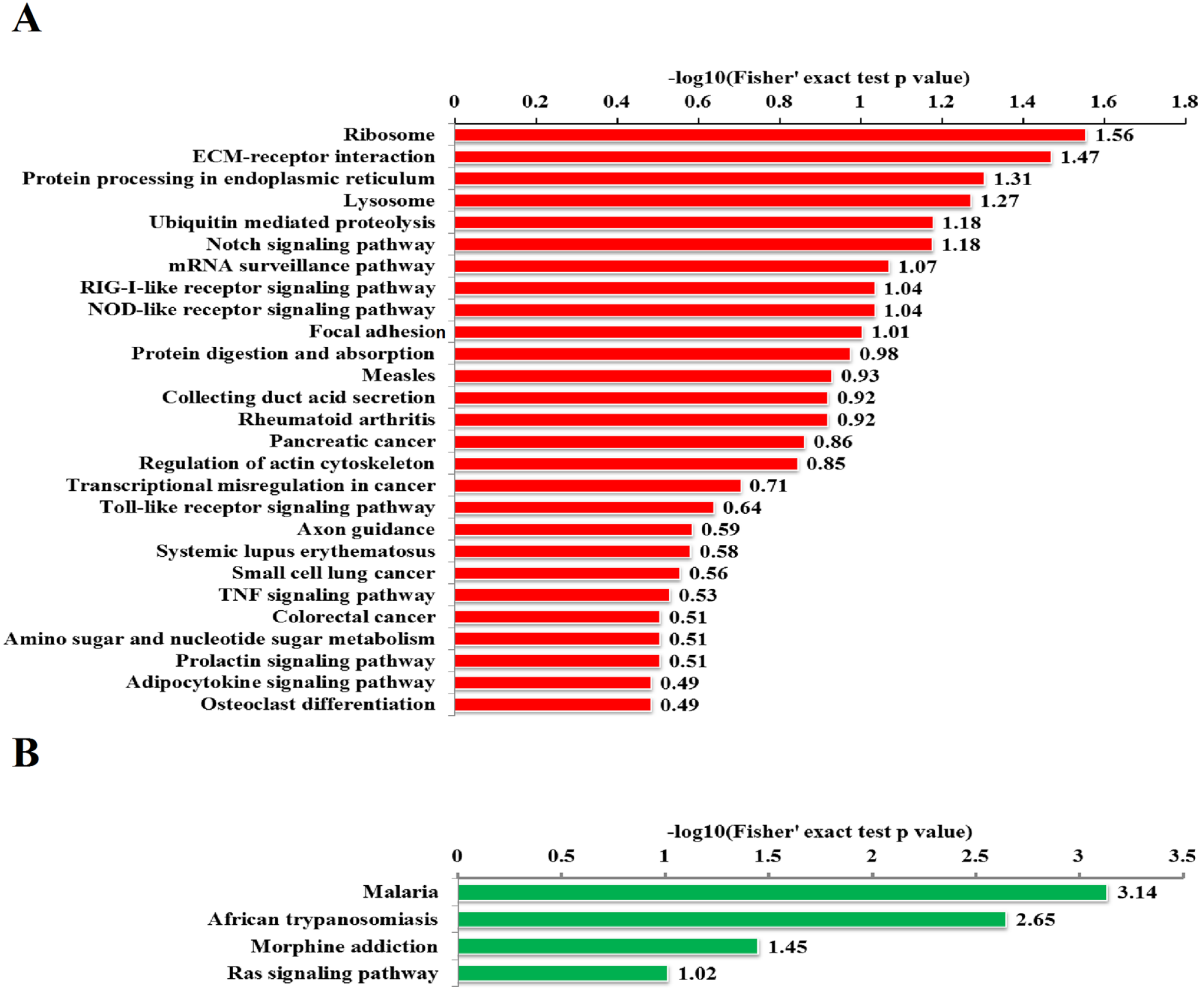
KEGG pathway-based enrichment analysis. (A) Pathway-based enrichment of upregulated proteins. (B) Pathway-based enrichment of downregulated proteins.

A KEGG pathway-based analysis was performed to identify pathways that were potentially affected by the differential protein expression between A30P*A53T transgenic and WT mice. A biological pathway analysis of common related proteins showed that the proteins were linked to a total of 31 KEGG pathways. The top three pathways from upregulated proteins were the ribosome, ECM–receptor interaction, and protein processing in the endoplasmic reticulum (ER) (Fig 5A). The top three pathways from downregulated proteins were malaria, African trypanosomiasis, and morphine addiction (Fig 5B). All KEGG pathway information and proteins involved are listed in S4 and S5 Tables. Several important proteins, including Q9DBY1, Q8R180, Q8BU14, Q61335, Q9WTU6, Q80UM7, P70362, and Q9Z2G6, with functions in protein export, protein degradation, and the ER, were classified as part of protein processing in the ER pathway (Fig 5, S4 Table).

### Validation of altered proteins

**Fig 6:**
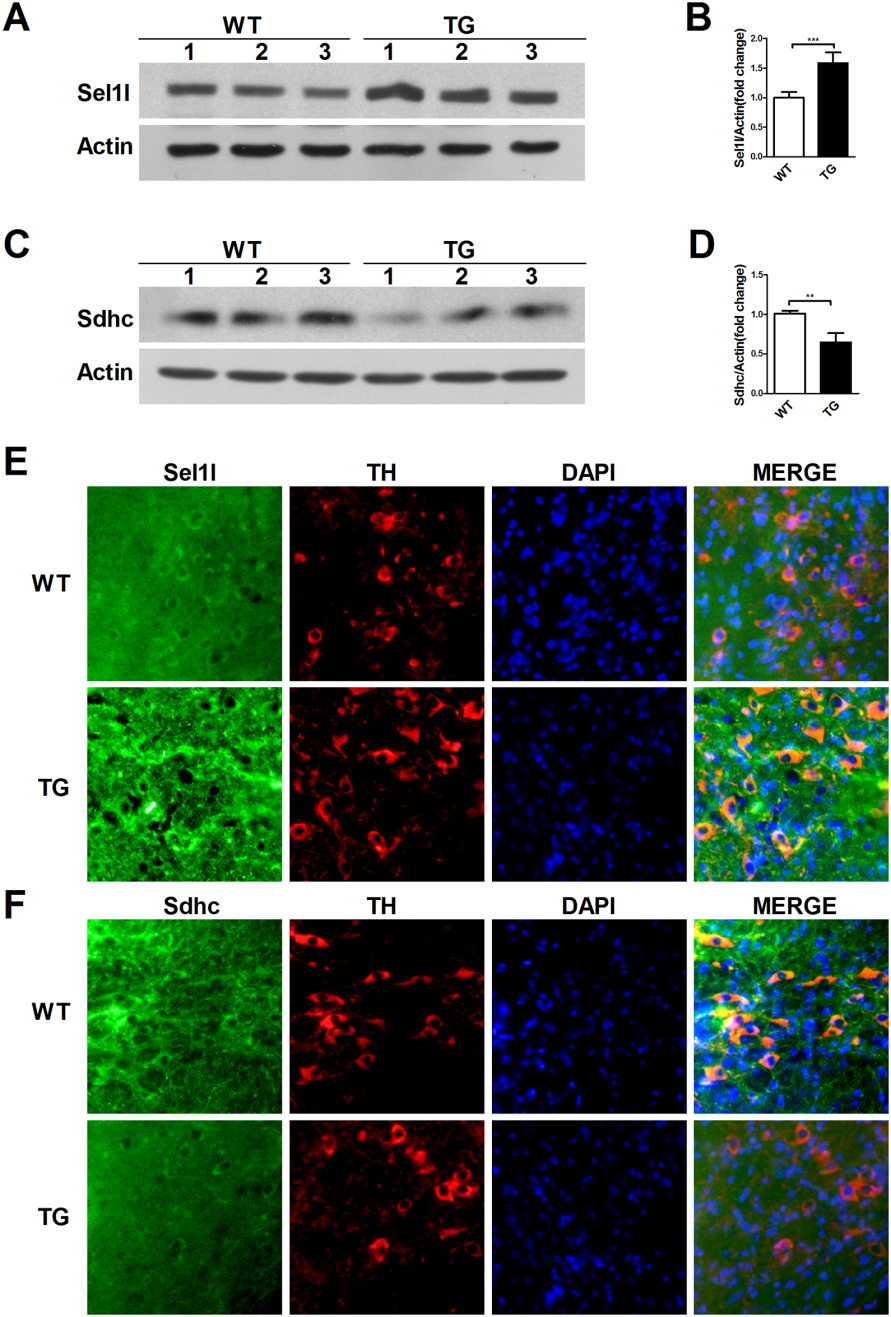
Validation of altered proteins in the SNpc tissue of TG mice and WT controls by western blotting and immunofluorescence staining. (A) The protein levels of Sel1l were detected by western blot in the SNpc tissue of TG mice and WT controls. (B) Quantification of the protein levels of Sel1l from (A) (n = 3). (C) The protein levels of Sdhc were measured by western blot in the SNpc tissue of TG mice and WT controls. (D) Quantification of the protein levels of Sdhc from (C) (n = 3). (E) Sel1l was monitored by dual immunolabeling of TH (red) and Sel1l (green) in the SNpc regions of TG mice and WT controls. (F) Sdhc was assessed by dual immunolabeling of TH (red) and Sdhc (green) in the SNpc regions of TG mice and WT controls.

To verify the reliability of the proteomics analysis, Sel1l and Sdhc were selected as representative proteins and subjected to western blotting, immunofluorescence staining and ELISA (Fig 6 and S1 Fig). The expression level of Sel1l in the SNpc of the TG group was higher than that in the WT group detected by western blotting and immunofluorescence staining (Fig 6A, 6B and 6E). The western blotting and immunofluorescence staining results showed that the expression level of Sdhc in the SNpc of the TG group was lower than in the WT group (Fig 6C, 6D and 6F). The results of western blotting and immunofluorescence staining for Sel1l and Sdhc using the SNpc of the WT and TG mice were consistent with those of the TMT proteomics. Additionally, the protein levels of Sel1l and Sdhc were not altered in the plasma of WT and TG mice monitored by ELISA (S1 Fig).

## Discussion

In this study, TMT-based quantitative proteomics analysis was applied to investigate the proteomic profiles and potential biomarkers in A30P*A53T α-synuclein transgenic mice, which may facilitate understanding the α-synuclein biology and pathological changes in synucleinopathies. We catalogued 4,715 proteins, which represent the larger proteome map to date. Over 249 proteins were upregulated with >1.2-fold change in A30P*A53T α-synuclein transgenic mice compared with WT mice. The top five upregulated proteins identified were the V-type proton ATPase 16 kDa proteolipid subunit (Atp6v0c), 6.8 kDa mitochondrial proteolipid, 60S ribosomal protein L27a, Transportin-2, and Protein sel-1 homolog 1 (Sel1l). Over 22 proteins were downregulated with <0.8-fold change. The top five downregulated proteins identified were Keratin, type I cytoskeletal 10; Succinate dehydrogenase cytochrome b560 subunit, mitochondrial (Sdhc); Rho GDP-dissociation inhibitor 2; Hyaluronan and proteoglycan link protein 2; and Poly (U)-binding-splicing factor PUF60. The validation results showed that the expression level of Sel1l and Sdhc in the SNpc of the TG group and WT group were consistent with those of the TMTs proteomics by western blotting and immunofluorescence staining. The top-ranking proteins closely associated with synucleinopathies, including Sel1l and Sdhc, may be involved in α-synuclein pathologies in synucleinopathies. Moreover, a biological KEGG pathway analysis of the altered proteins revealed 31 altered pathways. The top-ranking pathways closely associated with synucleinopathies included protein processing in the ER pathways.

A bioinformatics analysis of altered proteins in α-synuclein transgenic mice aids in the understanding of synucleinopathy complexity and the identification of early biomarkers. The top upregulated protein in this study was Atp6v0c, the bafilomycin A1-binding subunit of vacuolar ATPase[13]. Transfection of adeno-associated viral vectors harboring Atp6v0c in the mouse caudate putamen enhanced the depolarization-induced overflow of endogenous DA and ameliorated motor defects in a PD mouse model[14, 15]. Another study showed that Atp6v0c knockdown significantly increased the basal levels of microtubule-associated protein light chain 3-II, α-synuclein high-molecular weight species, and APP C-terminal fragments as well as inhibited autophagic flux[15]. These studies suggest that Atp6v0c may be involved in dopamine release from nerve terminals in the striatum of mice and that Atp6v0c may be helpful as a rescue molecule for gene therapy. Sel1l is a component of the ER stress degradation system and changed dramatically in our study. Omura et al. reported that hypodense zonisamide (an antiepileptic agent) improved the cardinal symptoms of PD by increasing Sel1l expression, thus inhibiting neuronal cell death in PD patients[16]. Sel1l knockout mice, with an average lifespan of 8–10 weeks, progressively developed motor dysfunction, including abnormal limb clasping and impaired gross and fine motor coordination[17]. These results indicate that Sel1l dysregulation or dysfunction may be involved in synucleinopathy pathogenesis. Sdhc is an important component of mitochondrial complex II and was obviously downregulated in our study. Ishii et al. reported that an Sdhc mutation resulted in increased reactive oxygen species production, leading to apoptosis and precocious aging in Caenorhabditis elegans[18, 19]. Sdhc ablation in mice resulted in a progressive dopaminergic neuron loss in the SNpc, and neurons were more susceptible to mitochondrial damage[18]. Thus, Sdhc mutations and Sdhc activity depression are strongly implicated in neuronal loss in synucleinopathies. Accordingly, Atp6v0c, Sel1l, and Sdhc are involved in alpha synuclein biology.

Molecular mechanisms underlying synucleinopathies are extensively studied; however, much remains unknown. Numerous studies have indicated that ER stress is involved in synucleinopathy pathogenesis[20–22]. The ER is central to protein folding in eukaryotic cells, and any perturbations altering ER homeostasis can result in the disruption of the folding process and accumulation of misfolded or unfolded proteins (i.e., ER stress) [20–22]. Under chronic ER stress, the accumulation of unfolded proteins and sustained unfolded protein response activates pro-apoptotic pathways and cell death, thereby eliminating damaged cells[20]. The ER accumulation of α-synuclein was observed in PD brain tissue[21]. Moreover, the accumulation and aggregation of misfolded/unfolded α-synuclein may promote sustained ER stress, resulting in the activation of death mechanisms in PD[20–22]. Here, we found that protein processing in the ER pathway was obviously altered, and Sel1l, a component of the ER stress degradation system, was significantly upregulated in the SNpc of α-synuclein transgenic mice, indicating that protein processing in the ER pathway is involved in synucleinopathies. Malaria and African trypanosomiasis, the top two pathways from downregulated proteins are meaningless, as they are based on the changes in hemoglobin and keratin proteins, which might be due to contamination with animal fur hair. The contamination is unavoidable in proteomics research although we strictly followed the operating instruction.

PD is the most prevalent of the synucleinopathies and has been the focus of much of the initial research on α-synuclein Lewy body pathology. The first motor symptoms of PD emerge approximately 5–30 years after actual disease onset[23]. Thus, PD therapies with dopamine agonists are used long after the number of nigrostriatal dopaminergic neurons required for normal regulation of motor functions is lost. Therefore, studies in this area searched for preclinical biomarkers that were easily accessible for analysis, including non-motor symptoms, changes in body fluid composition, and specific gene and protein expression in blood cells[23–25]. However, no obvious biomarkers were identified. Fortunately, technological advances have facilitated new assessments of disease pathology (e.g., proteomic profiling). Proteomics were employed to further our understanding of PD pathological mechanisms. Recent proteomic profiling studies in PD have investigated protein changes in the cerebrospinal fluid, plasma, and brain tissue of PD patients and in the brain tissue of MPTP- and 6-OHDA-treated animal models[26, 27]. Proteomic research in PD patients and animal models has shown that overt motor symptoms provide little help in the identification of preclinical biomarkers and preventive therapies for PD.

Therefore, in this study, we first used TMTs to identify differentially expressed proteins in the SNpc of A30P*A53T α-synuclein transgenic mice, which did not show overt phenotypes of PD. Here, we identified altered proteins for the early and specific detection of α-synuclein pathologies in synucleinopathies. Our findings provide information on the disease pathogenesis and etiology and clues for new therapeutic targets for synucleinopathies.

## Supporting information

S1 Checklist NC3Rs ARRIVE guidelines checklist. (DOCX)

S1 Fig Validation of altered proteins in the plasma of TG mice and WT controls by ELISA. (A) The protein levels of Sel1l were assessed by ELISA in the plasma of TG mice and WT controls (n = 6). (B) The protein levels of Sdhc were detected by ELISA in the plasma of TG mice and WT controls (n = 6).
(TIF)

S2 Fig Altered proteins in the cortical areas of TG mice and WT controls were detected by immunofluorescence staining. (A) Sel1l was monitored by immunolabeling of Sel1l (green) in the cortical areas of TG mice and WT controls. (B) Sdhc was assessed by immunolabeling of Sdhc (green) in the cortical areas of TG mice and WT controls.
(JPG)

S1 Table Summary of identified and quantified proteins and peptides. (XLSX)

S2 Table The GO distribution of upregulated proteins. (XLSX)

S3 Table The GO distribution of downregulated proteins. (XLSX)

S4 Table KEGG Pathway obtained from the upregulated proteins. (XLSX)

S5 Table KEGG Pathway obtained from the downregulated proteins. (XLSX)

## References

[1] Kim WS, Kagedal K, Halliday GM. Alpha-synuclein biology in Lewy body diseases. Alzheimer's research & therapy. 2014;6(5):73 doi: 10.1186/s13195-014-0073-2 ; 25580161 10.1186/s13195-014-0073-2 PMC4288216

[2] Burre J, Sharma M, Sudhof TC. Cell Biology and Pathophysiology of alpha-Synuclein. Cold Spring Harbor perspectives in medicine. 2017 doi: 10.1101/cshperspect.a024091 . 28108534 10.1101/cshperspect.a024091 PMC5519445

[3] Chung CY, Khurana V, Yi S, Sahni N, Loh KH, Auluck PK, et al In Situ Peroxidase Labeling and Mass-Spectrometry Connects Alpha-Synuclein Directly to Endocytic Trafficking and mRNA Metabolism in Neurons. Cell systems. 2017 doi: 10.1016/j.cels.2017.01.002 . 28131823 10.1016/j.cels.2017.01.002 PMC5578869

[4] Ingelsson M. Alpha-Synuclein Oligomers-Neurotoxic Molecules in Parkinson's Disease and Other Lewy Body Disorders. Frontiers in neuroscience. 2016;10:408 doi: 10.3389/fnins.2016.00408 ; 27656123 10.3389/fnins.2016.00408 PMC5011129

[5] Dehay B, Fernagut PO. Alpha-synuclein-based models of Parkinson's disease. Revue neurologique. 2016;172(6–7):371–8. doi: 10.1016/j.neurol.2016.04.003 . 27158042 10.1016/j.neurol.2016.04.003

[6] Acosta SA, Tajiri N, de la Pena I, Bastawrous M, Sanberg PR, Kaneko Y, et al Alpha-synuclein as a pathological link between chronic traumatic brain injury and Parkinson's disease. Journal of cellular physiology. 2015;230(5):1024–32. doi: 10.1002/jcp.24830 ; 25251017 10.1002/jcp.24830 PMC4328145

[7] Prasad K, Tarasewicz E, Strickland PA, O'Neill M, Mitchell SN, Merchant K, et al Biochemical and morphological consequences of human alpha-synuclein expression in a mouse alpha-synuclein null background. The European journal of neuroscience. 2011;33(4):642–56. doi: 10.1111/j.1460-9568.2010.07558.x ; 21272100 10.1111/j.1460-9568.2010.07558.x PMC3072281

[8] Thiruchelvam MJ, Powers JM, Cory-Slechta DA, Richfield EK. Risk factors for dopaminergic neuron loss in human alpha-synuclein transgenic mice. The European journal of neuroscience. 2004;19(4):845–54. Epub 2004/03/11. . 15009131 10.1111/j.0953-816x.2004.03139.x

[9] Richfield EK, Thiruchelvam MJ, Cory-Slechta DA, Wuertzer C, Gainetdinov RR, Caron MG, et al Behavioral and neurochemical effects of wild-type and mutated human alpha-synuclein in transgenic mice. Experimental neurology. 2002;175(1):35–48. doi: 10.1006/exnr.2002.7882 . 12009758 10.1006/exnr.2002.7882

[10] Pal R, Larsen JP, Moller SG. The Potential of Proteomics in Understanding Neurodegeneration. International review of neurobiology. 2015;121:25–58. doi: 10.1016/bs.irn.2015.05.002 . 26315761 10.1016/bs.irn.2015.05.002

[11] Ozgul S, Kasap M, Akpinar G, Kanli A, Guzel N, Karaosmanoglu K, et al Linking a compound-heterozygous Parkin mutant (Q311R and A371T) to Parkinson's disease by using proteomic and molecular approaches. Neurochemistry international. 2015;85–86:1–13. doi: 10.1016/j.neuint.2015.03.007 . 25865804 10.1016/j.neuint.2015.03.007

[12] Wang Q, Qian L, Chen SH, Chu CH, Wilson B, Oyarzabal E, et al Post-treatment with an ultra-low dose of NADPH oxidase inhibitor diphenyleneiodonium attenuates disease progression in multiple Parkinson's disease models. Brain: a journal of neurology. 2015;138(Pt 5):1247–62. doi: 10.1093/brain/awv034 ; 25716193 10.1093/brain/awv034 PMC4407187

[13] Jin D, Muramatsu S, Shimizu N, Yokoyama S, Hirai H, Yamada K, et al Dopamine release via the vacuolar ATPase V0 sector c-subunit, confirmed in N18 neuroblastoma cells, results in behavioral recovery in hemiparkinsonian mice. Neurochemistry international. 2012;61(6):907–12. doi: 10.1016/j.neuint.2011.12.021 . 22265874 10.1016/j.neuint.2011.12.021

[14] Higashida H, Yokoyama S, Tsuji C, Muramatsu SI. Neurotransmitter release: vacuolar ATPase V0 sector c-subunits in possible gene or cell therapies for Parkinson's, Alzheimer's, and psychiatric diseases. The journal of physiological sciences: JPS. 2017;67(1):11–7. doi: 10.1007/s12576-016-0462-3 . 27289535 10.1007/s12576-016-0462-3 PMC10717279

[15] Mangieri LR, Mader BJ, Thomas CE, Taylor CA, Luker AM, Tse TE, et al ATP6V0C knockdown in neuroblastoma cells alters autophagy-lysosome pathway function and metabolism of proteins that accumulate in neurodegenerative disease. PloS one. 2014;9(4):e93257 doi: 10.1371/journal.pone.0093257 ; 24695574 10.1371/journal.pone.0093257 PMC3973706

[16] Saltini G, Dominici R, Lovati C, Cattaneo M, Michelini S, Malferrari G, et al A novel polymorphism in SEL1L confers susceptibility to Alzheimer's disease. Neuroscience letters. 2006;398(1–2):53–8. doi: 10.1016/j.neulet.2005.12.038 . 16412574 10.1016/j.neulet.2005.12.038

[17] Omura T, Asari M, Yamamoto J, Kamiyama N, Oka K, Hoshina C, et al HRD1 levels increased by zonisamide prevented cell death and caspase-3 activation caused by endoplasmic reticulum stress in SH-SY5Y cells. Journal of molecular neuroscience: MN. 2012;46(3):527–35. doi: 10.1007/s12031-011-9638-8 . 21892618 10.1007/s12031-011-9638-8

[18] Diaz-Castro B, Pintado CO, Garcia-Flores P, Lopez-Barneo J, Piruat JI. Differential impairment of catecholaminergic cell maturation and survival by genetic mitochondrial complex II dysfunction. Molecular and cellular biology. 2012;32(16):3347–57. doi: 10.1128/MCB.00128-12 ; 22711987 10.1128/MCB.00128-12 PMC3434551

[19] Ishii N, Ishii T, Hartman PS. The role of the electron transport SDHC gene on lifespan and cancer. Mitochondrion. 2007;7(1–2):24–8. doi: 10.1016/j.mito.2006.11.012 . 17321223 10.1016/j.mito.2006.11.012

[20] Mercado G, Castillo V, Soto P, Sidhu A. ER stress and Parkinson's disease: Pathological inputs that converge into the secretory pathway. Brain Res. 2016;1648(Pt B):626–32. doi: 10.1016/j.brainres.2016.04.042 . 27103567 10.1016/j.brainres.2016.04.042

[21] Michel PP, Hirsch EC, Hunot S. Understanding Dopaminergic Cell Death Pathways in Parkinson Disease. Neuron. 2016;90(4):675–91. doi: 10.1016/j.neuron.2016.03.038 . 27196972 10.1016/j.neuron.2016.03.038

[22] Mercado G, Castillo V, Vidal R, Hetz C. ER proteostasis disturbances in Parkinson's disease: novel insights. Frontiers in aging neuroscience. 2015;7:39 doi: 10.3389/fnagi.2015.00039 ; 25870559 10.3389/fnagi.2015.00039 PMC4376001

[23] Caviness JN. Presymptomatic Parkinson's disease: The Arizona experience. Parkinsonism & related disorders. 2012;18:S203–S6. doi: 10.1016/s1353-8020(11)70063-3 22166435 10.1016/S1353-8020(11)70063-3

[24] Smith LM, Parr-Brownlie LC, Duncan EJ, Black MA, Gemmell NJ, Dearden PK, et al Striatal mRNA expression patterns underlying peak dose L-DOPA-induced dyskinesia in the 6-OHDA hemiparkinsonian rat. Neuroscience. 2016;324:238–51. doi: 10.1016/j.neuroscience.2016.03.012 . 26968766 10.1016/j.neuroscience.2016.03.012

[25] Glaab E, Schneider R. Comparative pathway and network analysis of brain transcriptome changes during adult aging and in Parkinson's disease. Neurobiology of disease. 2015;74:1–13. doi: 10.1016/j.nbd.2014.11.002 . 25447234 10.1016/j.nbd.2014.11.002

[26] Lin X, Shi M, Masilamoni JG, Dator R, Movius J, Aro P, et al Proteomic profiling in MPTP monkey model for early Parkinson disease biomarker discovery. Biochimica et biophysica acta. 2015;1854(7):779–87. doi: 10.1016/j.bbapap.2015.01.007 ; 25617661 10.1016/j.bbapap.2015.01.007 PMC4760626

[27] Wang ES, Yao HB, Chen YH, Wang G, Gao WW, Sun YR, et al Proteomic analysis of the cerebrospinal fluid of Parkinson's disease patients pre- and post-deep brain stimulation. Cellular physiology and biochemistry: international journal of experimental cellular physiology, biochemistry, and pharmacology. 2013;31(4–5):625–37. doi: 10.1159/000350082 . 23652646 10.1159/000350082

