# Supplementary material for: Quantitative proteomics in A30P*A53T α-synuclein transgenic mice reveals upregulation of Sel1l
Source: PLoS One. 2017 Aug 3;12(8):e0182092. doi: 10.1371/journal.pone.0182092 (PMC5542467; doi:10.1371/journal.pone.0182092)

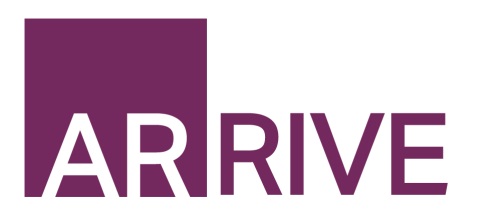


The ARRIVE Guidelines Checklist

Animal Research: Reporting In Vivo Experiments

Jianguo Yan1,2,3, Pei Zhang1,2,3, Fengjuan Jiao1,2,3, Qingzhi Wang1,2,3, Feng He1,2,3, Qian Zhang1,2,3, Zheng Zhang1,2,3, Zexi Lv1,2,3, Xiang Peng1,2,3, Hongwei Cai1,2,3, Bo Tian1,2,3*

*1 Department of Neurobiology, Tongji Medical School, Huazhong University of Science and Technology, 13 Hangkong Road, Wuhan, Hubei Province, 430030, P. R. China, 2 Key Laboratory of Neurological Diseases, Ministry of Education, 13 Hangkong Road, Wuhan, Hubei Province, 430030, P. R. China, 3 Institute for Brain Research, Collaborative Innovation Center for Brain Science, Huazhong University of Science and Technology, 13 Hangkong Road, Wuhan, Hubei Province, 430030, P. R. China.*

|  | | ITEM | RECOMMENDATION | Section/ Paragraph |
| --- | --- | --- | --- | --- |
| 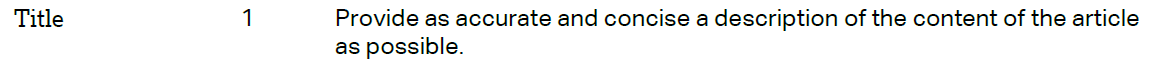 | | | Quantitative Proteomics in A30P*A53T α-synuclein Transgenic Mice Reveals Upregulation of Sel1l |  |
| 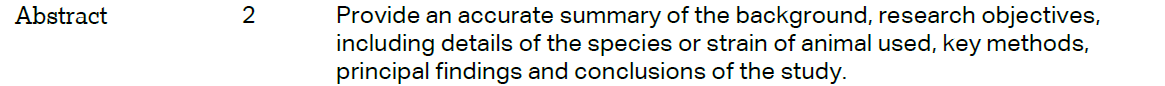 | | | BACKGROUND AND PURPOSE: α-Synuclein is an abundantly expressed neuronal protein that is at the center of focus in understanding a group of neurodegenerative disorders called synucleinopathies, which are characterized by the presence of aggregated α-synuclein intracellularly. Parkinson's disease (PD) is the most prevalent of the synucleinopathies and much of the initial research on α-synuclein Lewy body pathology. However, the mechanism of α-synuclein biology in PD pathogenesis is not fully understood.  EXPERIMENTAL APPROACH: Mice overexpressing human A30P*A53T α-synuclein were evaluated by motor behavior test and TH positive neurons count, then the two-dimensional liquid chromatography-tandem mass spectrometry coupled with tandem mass tags (TMTs) labeling was employed to quantitatively identify the differentially expressed proteins of substantia nigra pars compacta (SNpc) tissue samples that obtained from theα-synuclein transgenic mice and wild type controls.  KEY RESULTS: The number of SNpc dopaminergic neurons and PD-related behavior were unchanged in A30P*A53T transgenic mice at the age of 6 months. Of 4,715 identified proteins by proteomic techniques, 271 were differentially expressed, including 249 upregulated and 22 downregulated proteins. These alterations were primarily associated with mitochondrial dysfunction, oxidative stress, ubiquitin-proteasome system impairment, and endoplasmic reticulum (ER) stress. Some drastically changed proteins, including Atp6v0c, SEL1L, and Sdhc, may be involved in α-synuclein biology in synucleinopathies, especially PD. Of 31 identified KEGG pathways, we focused on protein processing in ER and morphine addiction pathways.  CONCLUSIONS AND IMPLICATIONS: Therefore, in this study, we first identified differentially expressed proteins in the SNpc of A30P*A53T α-synuclein transgenic mice using TMTs. Here we identified novel biomarkers for early and specific detection of α-synuclein pathologies in synucleinopathies. Our findings provide information on the disease pathogenesis and etiology and clues for new therapeutic targets for synucleinopathies, especially PD. |  |
| INTRODUCTION | | |  |  |
| 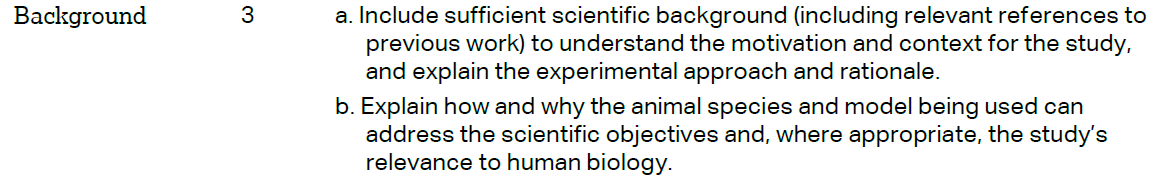 | | | α-Synuclein is an abundantly expressed neuronal protein that is at the center of focus in understanding a group of neurodegenerative disorders called synucleinopathies, which are characterized by the presence of aggregated α-synuclein intracellularly. Parkinson's disease (PD) is the most prevalent of the synucleinopathies and much of the initial research on α-synuclein Lewy body (LBs) pathology [1]. LBs are mainly composed of insoluble α-synuclein and ubiquitinylated proteins[2]. The numerous murine transgenic lines overexpressing human WT, A53T, or A30P mutant α-synuclein develop synucleinopathy, neurodegeneration, loss of striatal dopamine, and locomotor dysfunction[1-3]. Since then, our understanding of the importance of α-synuclein biology in PD pathogenesis has grown considerably. However, the mechanisms that underlie the aberrant functions of α-synuclein and how these impact on disease pathogenesis remain poorly understood. Proteomics is a powerful methodology to investigate how protein expression is affected in the pathogenesis of a disease process, providing a complement to the information obtained by functional genomics.The overexpression of human A30P*A53T α-synuclein in mice is widely used in PD research[4-6]. The number of SNpc dopaminergic neurons and levels of dopamine were unchanged in A30P*A53T transgenic mice up to 9 months old[4], but significantly decreased levels of dopamine and motor impairment were recognized at 16 months old[5].Therefore, the A30P*A53T α-synuclein transgenic mice is a useful model for analyzing the pathological cascade from aggregated α-synuclein to motor disturbance. |  |
| 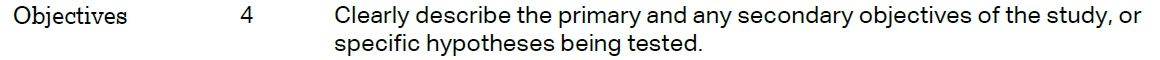 | | | In order to gain insight into the mechanism of α-synuclein biology in synucleinopathies, especially PD, we used TMTs to generate comparative protein profiles of SNpc samples obtained from A30P*A53T α-synuclein transgenic mice and controls at the age of 6 months. We compared SNpc tissue levels of candidate proteins to evaluate their ability to discriminate between α-synuclein transgenic and control mice. These findings indicate that proteomics is a useful method to investigate the crucial pathogenesis of α-synucleinopathies, and it may help to develop therapeutic targets. |  |
| METHODS | | |  |  |
| 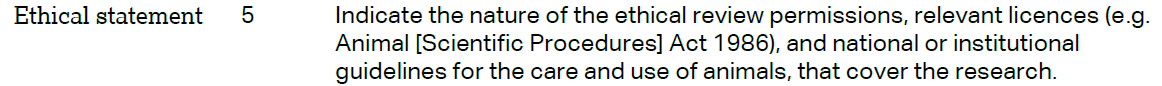 | | | The protocol was approved by the Animal Care and Use Committee of Huazhong University of Science and Technology. |  |
| 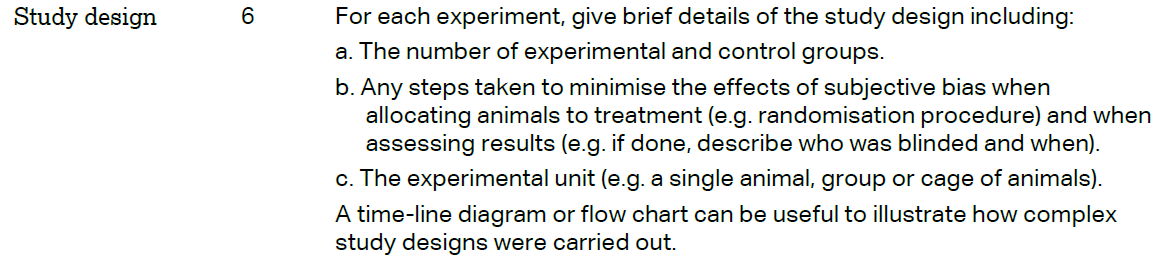 | | | The A30P*A53T α-synuclein transgenic mice and its wild type littermate at the age of 6 months were investigated by rotarod test (n=15), grip strength test (n=15), TH positive neurons count (n=3) and proteomic analyses (n=40).  The results of the experiment were taken by blind statistics to minimise the effects of subjective bias.  The experimental unit is a single animal. |  |
| 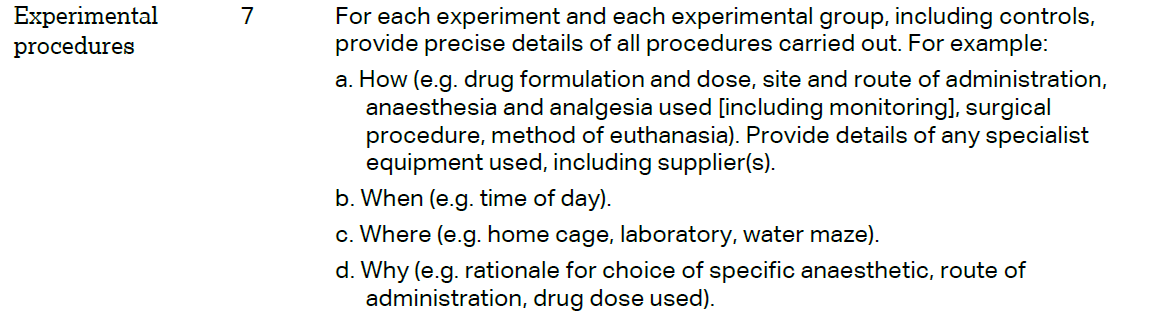 | | | The rotarod test was performed to assess motor coordination by placing mice on a rotating rod that runs at an accelerating speed from 3 to 30 rpm over a 5-min period. If a mouse falls onto an underlying platform, the detector automatically stops and records the fall down latency. The forelimb grip strength test was used to evaluate the muscle strength or neuromuscular activity in mice. The mice were held up to grip the pull bar on the grip wire with only their front paws was steadily pulled back until they could not hold on any longer. After anesthetized, the brains were removed from the skull, and fixed in 4% paraformaldehyde overnight, then stored at 4 °C in 30% sucrose solution until they sank. Brains were frozen sectioned using a sliding microtome (Leica, Germany) into 30 μm coronal sections. For immunofluorescence staining, the slices were permeabilized in 0.3% triton for 10 min, then blocked with 10 % serum in PBS for 1 h and incubated with a primary antibody against tyrosine hydroxylase (TH) (Santa Cruz, sc-374047, 1:200) overnight at 4 °C. The next day, Dylight 594-Conjugated AffiniPure Goat Anti-Mouse IgG secondary antibodies were added to the sections. Nuclei were stained by DAPI. The slices were imaged using a microscopy (Leica DFC320, Germany). The number of TH-immunoreactive positive neurons in the SNpc was counted. The SNpc tissues of the transgenic mice and its wild type littermate at the age of 6 months were removed, and samples were frozen immediately in liquid nitrogen. Approximately 100 μg protein for each sample was digested with trypsin for the TMTs experiments.  All behavioral experiments were performed in the behavioral laboratory According to the protocol. |  |
| 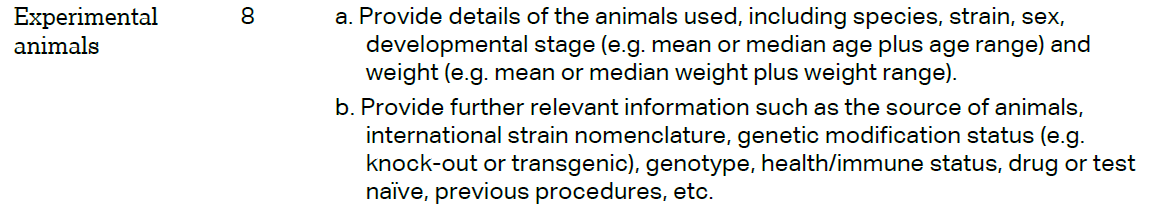 | | | C57BL/6J-Tg (Th-SNCA*A30P*A53T) 39Eric/J transgenic mic (25 ± 1 g, n = 43) and wild type littermate (25 ± 1 g, n = 43), aged 6 months s, were used.  To establish a transgenic PD mouse model, C57BL/6J-Tg (Th-SNCA*A30P*A53T) 39Eric/J transgenic mice (Stock number: 008239) were purchased from the Jackson Laboratory. To maintain the α-synuclein-A30P*A53T transgenic (TG) mice in our lab, established transgenic mice were mated with wild type C57BL/6J background (Beijing Vital River Laboratory Animal Technology Co., Ltd, Beijing, China). Offspring were genotyped by PCR of tail-tip DNA according to the genotyping protocols database of the Jackson Laboratory website. Animals were housed under standard housing conditions 12h light/12h dark cycle, with unlimited access to water and chow. |  |

The ARRIVE guidelines. Originally published in *PLoS Biology*, June 2010^1^

| 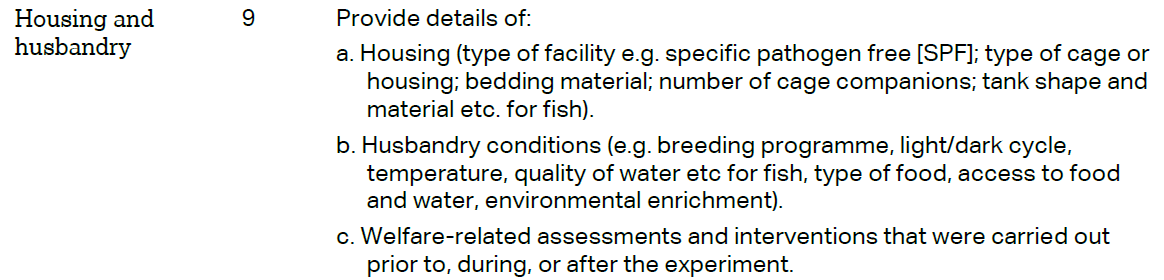 | Animals were housed under standard housing conditions 12h light/12h dark cycle with lights on at 8:30pm in a temperature (25±1ºC) and humidity (55±5%) controlled room (SPF). Prior to surgery the animals were housed in cages filled with hygiene animal beddin enriched with next boxes .  All mice were allowed free access to water and a maintenance diet. | |
| --- | --- | --- |
| 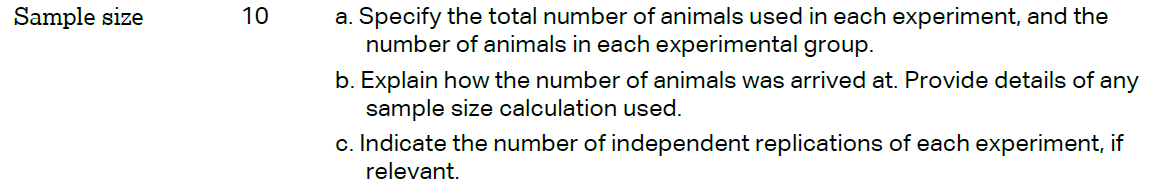 | The A30P*A53T α-synuclein transgenic mice(n=43) and its wild type littermate (n=43) at the age of 6 months were investigated by rotarod test (n=15), grip strength test (n=15), TH positive neurons count (n=3) and proteomic analyses (n=40).  Sample size calculations were performed in STATA/IC 10 (StataCorp,College Station, Texas, USA) with the sampsi function. | |
| 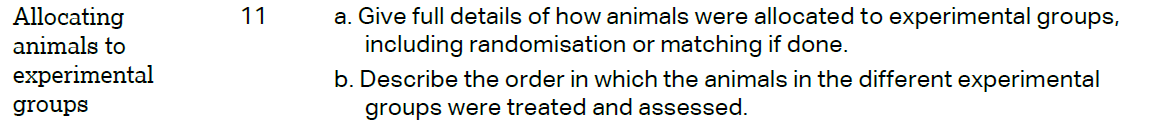 | Transgenic mic and wild type littermate were allocated equally according to matched-pair grouping design.  Mice were investigated by rotarod test first, grip strength test second, TH positive neurons count third and proteomic analyses last. | |
| 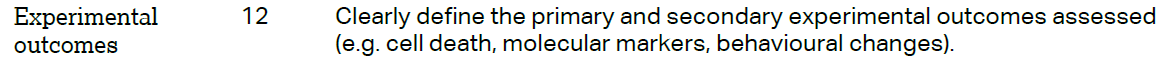 | Some drastically changed proteins, including Atp6v0c, SEL1L, and Sdhc, may be involved in α-synuclein biology in in synucleinopathies, especially PD. Our findings suggest that these identified proteins may function as biomarkers for in synucleinopathies and serve as novel therapeutic targets. | |
| 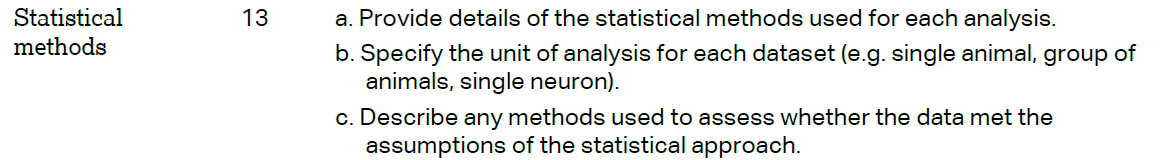 | All results were analyzed by two-tailed unpaired t-tests. All data are presented as the mean ± SD. All results are representative of at least three independent experiments. P value less than 0.05 was considered to be statistically significant. | |
| RESULTS |  | |
| 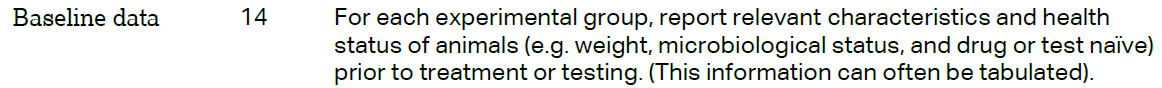 | The animals’ health status was monitored throughout the experiments by a health surveillance programme. The mice were free of all viral, bacterial, and parasitic pathogens listed in the recommendations. | |
| 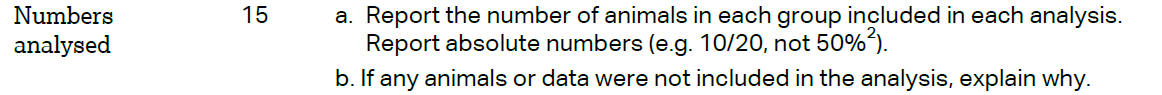 | C57BL/6J-Tg (Th-SNCA*A30P*A53T) 39Eric/J transgenic mic (25 ± 1 g, n = 43) and wild type littermate (25 ± 1 g, n = 43), aged 6 months s, were used. | |
| 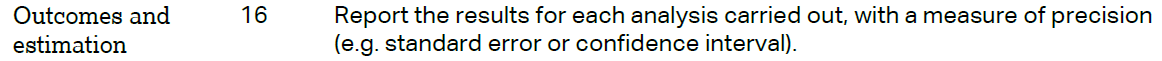 | In accordance with the ARRIVE guidelines (Kilkenny et al. 2010), we have reported measures of precision, confidence, and n to provide an indication of significance. | |
| 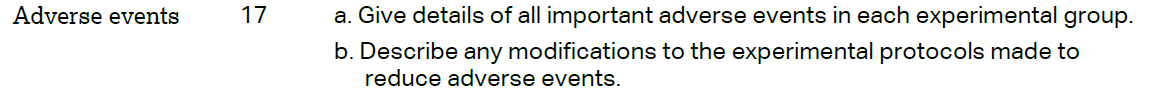 | Grip strength test is not accurate each time. So, we measured 10 times per mouse, the maximal force (in grams) measurement was recorded. | |
| DISCUSSION |  | |
| 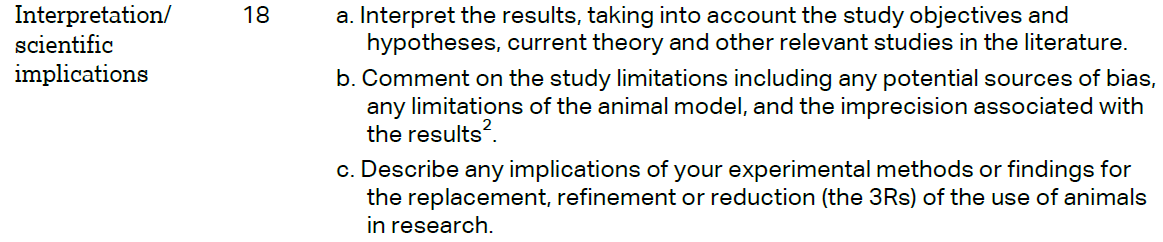 | The insoluble aggregated α-synuclein is widely recognized as a critical component of the progressive neurodegeneration of synucleinopathies, especially PD. However, the specific mechanism of α-synuclein biology in synucleinopathies is not fully understood. In this study, mice overexpressing human A30P*A53T α-synuclein were evaluated by motor behavior test and TH positive neurons count, then the two-dimensional liquid chromatography-tandem mass spectrometry coupled with tandem mass tags (TMTs) labeling was employed to quantitatively identify the differentially expressed proteins of substantia nigra pars compacta (SNpc) tissue samples that obtained from theα-synuclein transgenic mice and wild type controls. Of 4,715 identified proteins by proteomic techniques, 271 were differentially expressed, including 249 upregulated and 22 downregulated proteins. Some drastically changed proteins, including Atp6v0c, SEL1L, and Sdhc, may be involved in α-synuclein pathologies of synucleinopathies, especially PD. Our findings suggest that these identified proteins may function as biomarkers for synucleinopathies diagnosis and serve as novel therapeutic targets.  A limitation of this study is the fact that our study is about the quantitative expression proteomics in the brains of overexpressing human A30P*A53T α-synuclein mice. A lot of findings suggest that phosphorylation changes in PD should been focused on. The next step is to investigate the phosphoproteomics profile in the SNpc tissue A30P*A53T transgenic mice.  The new apparatus shows potential for considerably reducing the number of animals used in PD research. | |
| 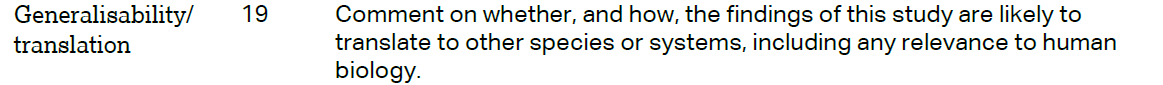 | Here we identified novel biomarkers for early and specific detection of α-synuclein pathologies in synucleinopathies by using TMTs quantitative proteomics in the SNpc of A30P*A53T α-synuclein transgenic mice. Our findings provide information on the disease pathogenesis and etiology and clues for new therapeutic targets for synucleinopathies, especially PD. | |
| 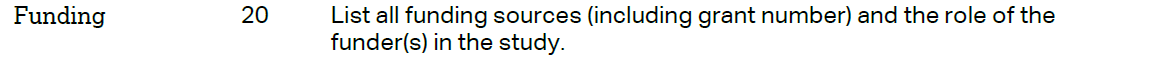 | | This work was supported financially by National Natural Science Foundation of China (No. 31371384, 31571044 to B.T. and No. 31600821 to P.Z.), Program for New Century Excellent Talents in University (No. NCET-10-0415 to B.T.), and China Postdoctoral Scientific Foundation (No. 2015M582226 to P.Z.). |


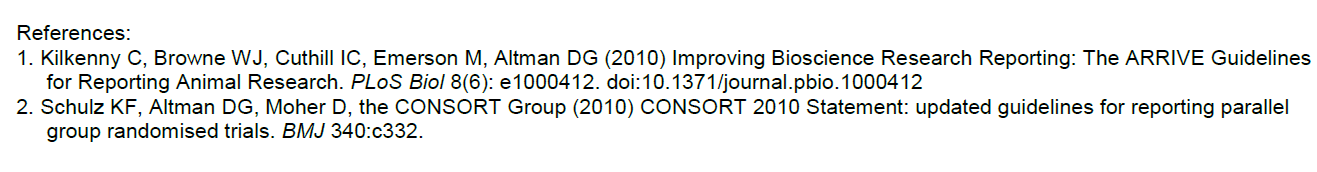


References

1. Ingelsson M. Alpha-Synuclein Oligomers-Neurotoxic Molecules in Parkinson's Disease and Other Lewy Body Disorders. Frontiers in neuroscience. 2016;10:408. doi: 10.3389/fnins.2016.00408. PubMed PMID: 27656123; PubMed Central PMCID: PMC5011129.

2. Dehay B, Fernagut PO. Alpha-synuclein-based models of Parkinson's disease. Revue neurologique. 2016;172(6-7):371-8. doi: 10.1016/j.neurol.2016.04.003. PubMed PMID: 27158042.

3. Acosta SA, Tajiri N, de la Pena I, Bastawrous M, Sanberg PR, Kaneko Y, et al. Alpha-synuclein as a pathological link between chronic traumatic brain injury and Parkinson's disease. Journal of cellular physiology. 2015;230(5):1024-32. doi: 10.1002/jcp.24830. PubMed PMID: 25251017; PubMed Central PMCID: PMC4328145.

4. Prasad K, Tarasewicz E, Strickland PA, O'Neill M, Mitchell SN, Merchant K, et al. Biochemical and morphological consequences of human alpha-synuclein expression in a mouse alpha-synuclein null background. The European journal of neuroscience. 2011;33(4):642-56. doi: 10.1111/j.1460-9568.2010.07558.x. PubMed PMID: 21272100; PubMed Central PMCID: PMC3072281.

5. Thiruchelvam MJ, Powers JM, Cory-Slechta DA, Richfield EK. Risk factors for dopaminergic neuron loss in human alpha-synuclein transgenic mice. The European journal of neuroscience. 2004;19(4):845-54. Epub 2004/03/11. PubMed PMID: 15009131.

6. Richfield EK, Thiruchelvam MJ, Cory-Slechta DA, Wuertzer C, Gainetdinov RR, Caron MG, et al. Behavioral and neurochemical effects of wild-type and mutated human alpha-synuclein in transgenic mice. Experimental neurology. 2002;175(1):35-48. doi: 10.1006/exnr.2002.7882. PubMed PMID: 12009758.
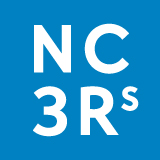

Supplement: S1 Checklist — (DOCX) [file pone.0182092.s001.docx]
