# Supplementary figures and images for: Quantitative proteomics in A30P*A53T α-synuclein transgenic mice reveals upregulation of Sel1l
Source: PLoS One. 2017 Aug 3;12(8):e0182092. doi: 10.1371/journal.pone.0182092 (PMC5542467; doi:10.1371/journal.pone.0182092)

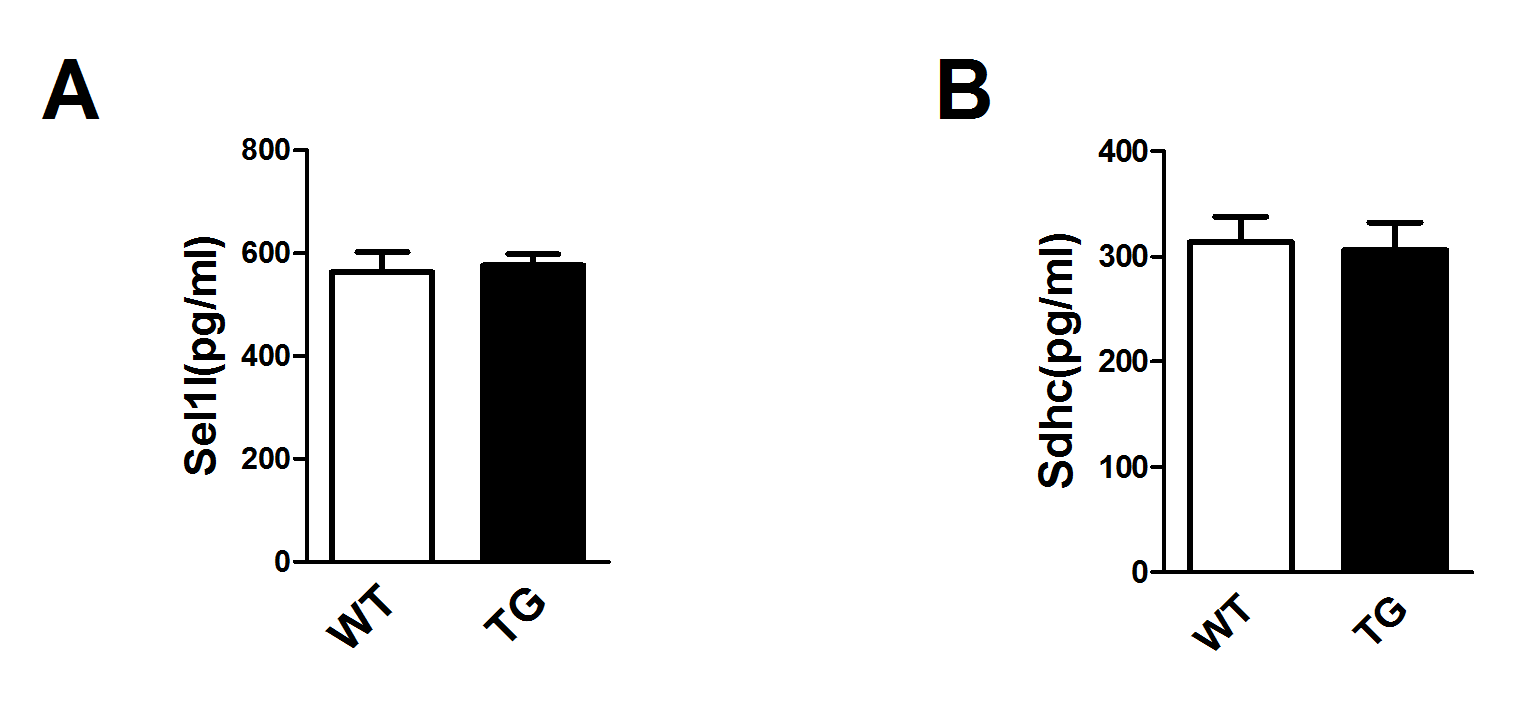

Supplement: S1 Fig — (A) The protein levels of Sel1l were assessed by ELISA in the plasma of TG mice and WT controls (n = 6). (B) The protein levels of Sdhc were detected by ELISA in the plasma of TG mice and WT controls (n = 6). (TIF) [file pone.0182092.s002.tif]

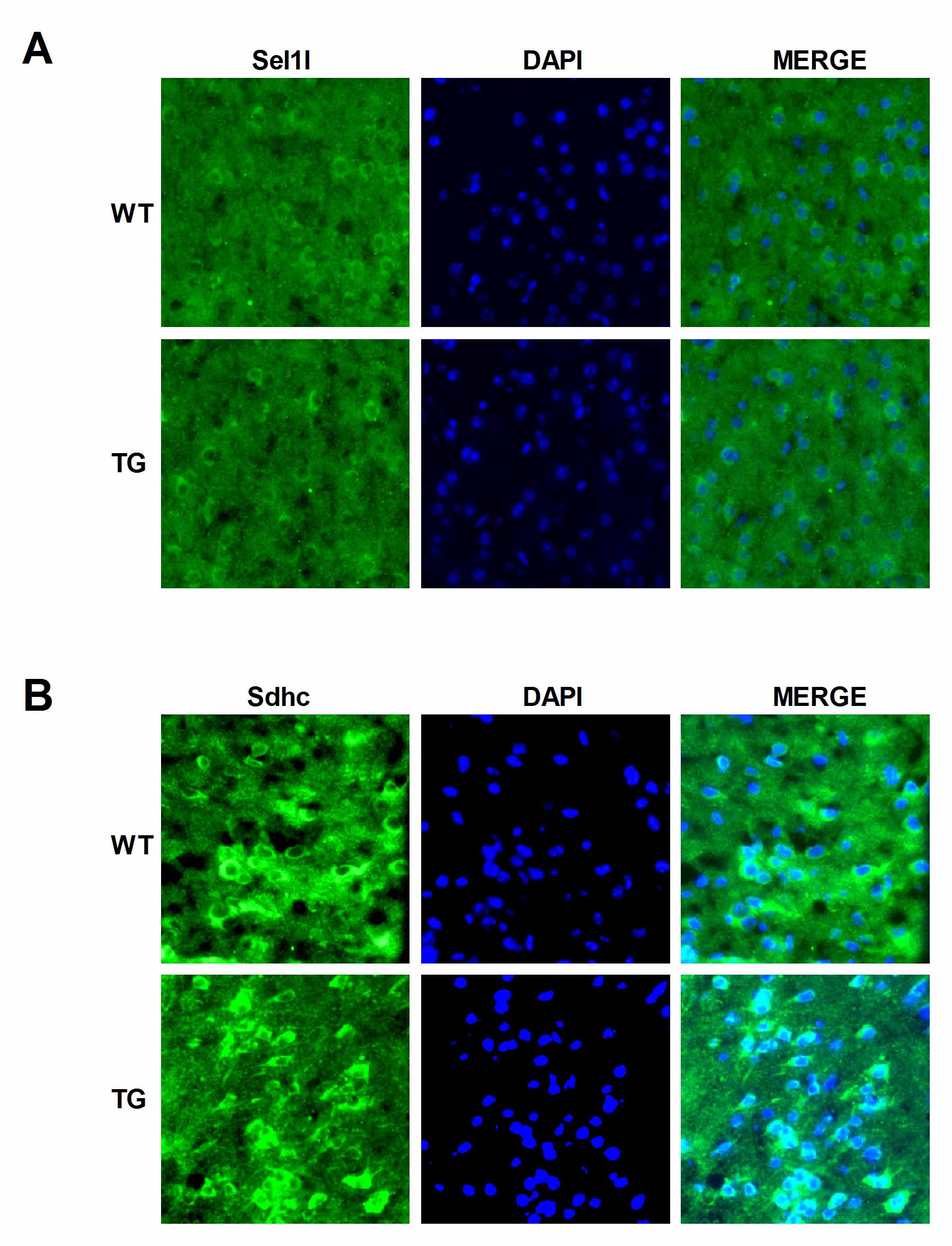

Supplement: S2 Fig — (A) Sel1l was monitored by immunolabeling of Sel1l (green) in the cortical areas of TG mice and WT controls. (B) Sdhc was assessed by immunolabeling of Sdhc (green) in the cortical areas of TG mice and WT controls. (JPG) [file pone.0182092.s003.jpg]
